# Morphological Variations in Toad‐Headed Agama: Potential Responses to Diverse Microhabitats

**DOI:** 10.1002/ece3.72188

**Published:** 2025-09-24

**Authors:** Shuran Li, Jingjing Chen, Xixi Liang, Xin Hao, Chenxu Wang, Baojun Sun, Yongpu Zhang

**Affiliations:** ^1^ College of Life and Environmental Science Wenzhou University Wenzhou Zhejiang China; ^2^ Chinese Academy of Medical Sciences & Peking Union Medical College Beijing China; ^3^ Key Laboratory of Animal Ecology and Conservation Biology, Institute of Zoology Chinese Academy of Sciences Beijing China

**Keywords:** adaptative strategy, desert, locomotion, microhabitat variation, morphological variation, soil matrix

## Abstract

The adaptive response of animals to microhabitat variations attracts much attention in evolutionary biology. Animal morphological traits exhibit close associations with microhabitat utilization, critically influencing organismal performance in specific habitats. However, how diverse desert microhabitats drive variations in morphology and related functions remains largely unclear. Here, with two populations of toad‐headed agama ( *Phrynocephalus przewalskii* ) inhabiting semi‐arid and arid areas, we compared the microhabitat characteristics as well as the variations in morphological characteristics and locomotor performances (running and burrowing) of lizards. Furthermore, by relating the functional differences and morphology, and thus the variation in microhabitats, we aimed to predict the adaptive morphological responses of toad‐headed agama to a variation in microhabitats. We found the population from open microhabitats with solid substrates possessed longer forelimbs (brachium and antebrachium) and appendages (metacarpus, phalanges, front claw, and hind claw). They also presented higher sprint speeds and enhanced burrowing capabilities, particularly on a hard soil matrix. Therefore, the differences in morphology and function between the populations support the hypothesis that morphological characteristics are compatible with function and potentially indicate adaptive strategies within different microhabitats.

## Graphical abstract

This paper employed two populations of toad‐headed lizards that inhabited semi‐arid and arid areas as the study system to assess their geographical variation in morphology and functions. We found that these population differences support the hypothesis that morphological characteristics are compatible with functions through experiments and data analysis. It potentially indicates that lizards have adaptive strategies to different substrates in microhabitats.

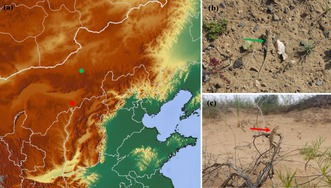

## 1 Introduction

The adaptive response of animals to local microhabitats is one of the central topics in ecology and evolution (Goodman et al. 2008; Kawecki and Ebert 2004; Yuan et al. 2020). Within the ecomorphological paradigm, morphological variation is thought to underpin differences in performance capacities, thereby driving fitness variation among individuals in specific habitats (Arnold 1983; Donihue et al. 2018; Melville and Swain 2000). These relationships are further modulated by abiotic factors (e.g., temperature) and biotic interactions (e.g., predation, competition) (Clusella‐Trullas et al. 2011; Dufour et al. 2018; Pringle et al. 2019). Interpopulation comparisons provide a powerful framework for disentangling these selective forces and revealing ecomorphological divergence at the intraspecific level, which could be predicted to detect either phenotypic plasticity or potential early steps toward fine‐scale microhabitat adaptations (Higham et al. 2015; Higham and Russell 2010; Sinervo and Losos 1991).

One of the predominant performance traits is locomotion, such as climbing, running, and burrowing, which can enhance survival through efficient foraging and predator escape, while facilitating reproductive success via mate and territorial competition (Garland Jr. et al. 1990; Husak 2006; Husak et al. 2006; Kinlaw 1999; Perry et al. 2004). For instance, faster lizards exhibit enhanced predator escape capability (Husak 2006) and a higher social dominance rank than slower conspecifics (Robson and Miles 2000). Furthermore, burrows serve not only as shelters from predators (Cloudsley‐Thompson 1991; Hildebrand 1985) but also as critical buffers against extreme temperatures and desiccation for numerous animals (Chen et al. 2021; Moore et al. 2018). Reptiles, as ectotherms, are particularly vulnerable to climatic extremes and serve as key models for studying microhabitat‐driven morphological evolution (Donihue 2016; Riedel et al. 2024; Yuan et al. 2019). Body and limb proportions in reptiles are considered essential factors in determining individual locomotor performance, and thus fitness. For example, it is well established that lizard running speed is positively correlated with limb length (Bonine and Garland 1999; Higham and Russell 2010; McElroy and Reilly 2009; Sinervo and Losos 1991; Tan et al. 2020). Moreover, lizards' sharp and curved claws can adhere to rough substrates, whereas fringed toes can move on soft and fine sand (Higham 2015; Pianka and Vitt 2003). In addition, tortoises and lizards with longer limbs or claws are hypothesized to provide higher impulse and possess enhanced burrowing ability; however, this relationship awaits empirical validation (Hildebrand 1985; Ribas et al. 2004; Teixeira‐Filho et al. 2001; Warner et al. 2006). Conversely, in head‐first burrowing serpentiform species, more elongated trunks and concave heads correlate with greater penetration force and accelerated burrowing speed (Bergmann and Berry 2021; de Barros et al. 2021). Nevertheless, functional tradeoffs can arise where selection pressure to improve one performance trait constrains the efficacy of others (Pasi and Carrier 2003; Simon et al. 2022; Vanhooydonck et al. 2001). For instance, a tradeoff between sprint speed and endurance occurs in many lizards as greater muscle mass improves sprinting but reduces stamina through higher energetic costs, while a fiber‐type tradeoff exists between fast‐twitch (sprint) and slow‐twitch (endurance) muscle fibers (Tan et al. 2020; Vanhooydonck et al. 2014; Vanhooydonck et al. 2001). Additionally, in Asian house geckos ( *Hemidactylus frenatus* ), males with larger heads enhance biting capacity at the expense of sprint performance (Cameron et al. 2013).

In phylogenetic research, the functions of specific morphological traits also vary depending on the microhabitat characteristics (Da Silva et al. 2014; Donihue 2016; Zheng et al. 2021). For instance, geckos inhabiting sparsely vegetated environments exhibit more elongated limb proportions than those from densely vegetated habitats (Zimin et al. 2024). Locomotor performance in lizards is further profoundly influenced by substrate structure (Naylor and Higham 2022; Tulli et al. 2012), such as sand‐dwelling lizards with fringed toes outperforming species lacking this trait in acceleration capacity on sandy substrates (Vanhooydonck et al. 2015). Moreover, in compacted soil habitats where substrates are harder and more difficult to excavate, worm lizards develop more tapered snouts and narrower cranial morphology to enhance burrowing capacity (Kirchner et al. 2025). Therefore, microhabitat divergence among lizard populations can drive divergent morphological and performance evolution, but intraspecific studies remain underrepresented (Higham et al. 2015).

As ectotherms, reptiles achieve high abundance and ecological dominance in arid and semi‐arid regions characterized by pronounced temperature fluctuations and persistent aridity (Roll et al. 2017). Within these extreme habitats, they have evolved morphological, physiological, and behavioral adaptations to withstand temperature and humidity extremes. Additionally, the structurally simplified desert environment further facilitates niche partitioning, as the vegetation generates heterogeneous thermal microhabitats, and sandy substrates provide essential refugia and nesting sites (Li et al. 2018; Zeng et al. 2016). Consequently, comparative studies of species/populations inhabiting these areas offer a suitable research model to elucidate the relationship between morphology, performance, and habitat (Higham 2015).

**FIGURE 1:**
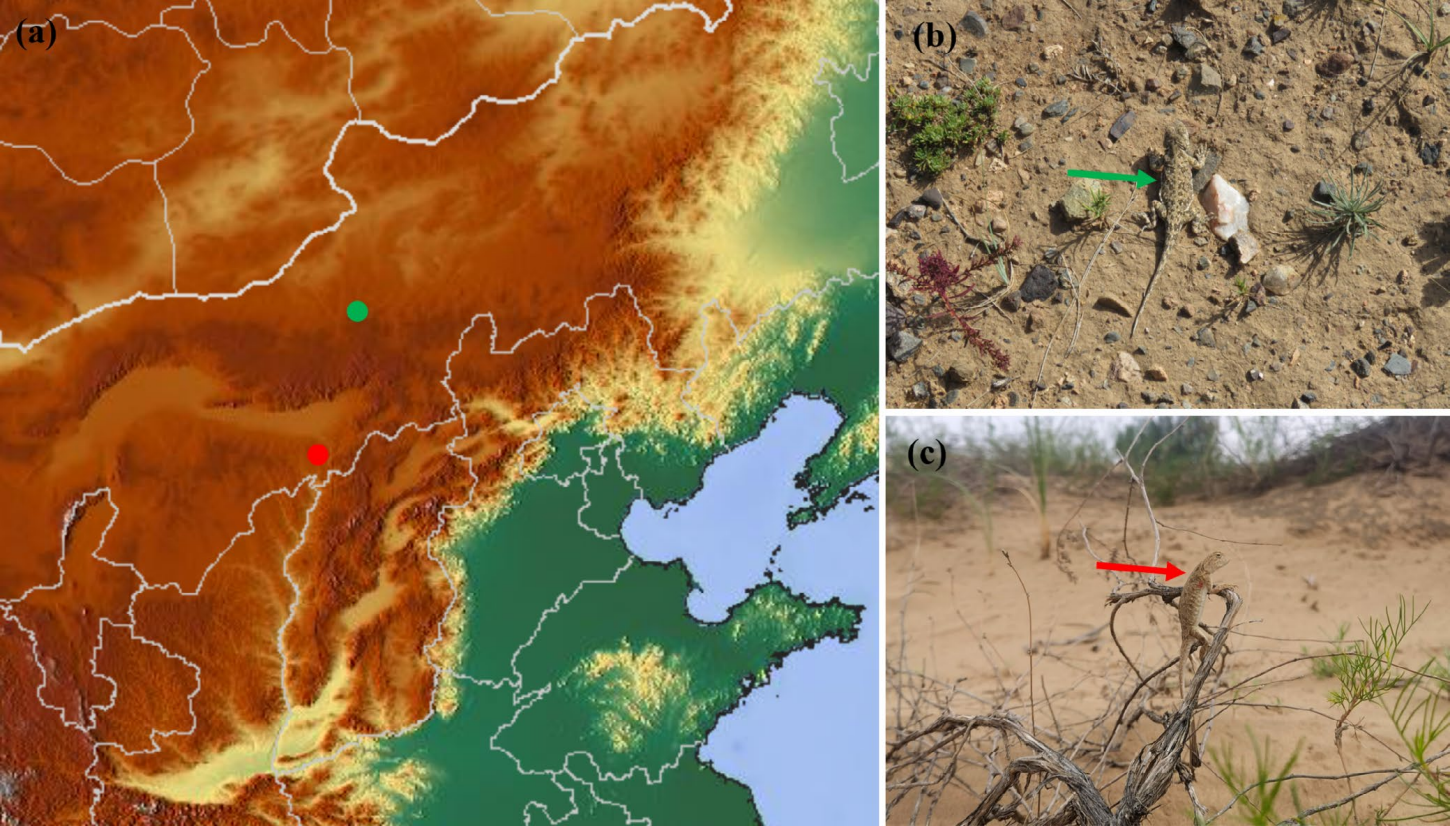
Locations of Gegentala and Shierliancheng populations of *Phrynocephalus przewalskii* and its microhabitat characteristics. (a) Study sites of Gegentala (green circle) and Shierliancheng (red circle). (b) Typical microhabitat of Gegentala; the green arrow points to the location of one individual lizard. (c) Typical microhabitat of Shierliancheng; the red arrow points to the location of one individual lizard.

The toad‐headed agama ( *Phrynocephalus przewalskii* ) is a small, desert‐dwelling lizard (snout–vent length [SVL] up to 60 mm), which is widely distributed in arid and semi‐arid areas in northern China (Zhao et al. 1999). The lizard digs its own burrows in the sand as shelters or nests by alternately scooping sand with fore and hind limbs (Chen et al. 2021; Li et al. 2018). The vegetation cover and soil matrix of two adjacent, conspecific populations in Gegentala and Shierliancheng are entirely different and significantly affected by climatic conditions (Figure 1). These two geographic areas are separated by the Yellow River and Yin Mountains, with a distance of ~250 km (Figure 1a), which may be potential barriers for gene flow between the two populations. The soil matrix of Gegentala is sandy loam soil and that of Shierliancheng is eolian sandy soil. The vegetation cover in Gegentala varies remarkably across seasons and is affected by rainfall; whereas in Shierliancheng, it is relatively stable across seasons. Therefore, the toad‐headed agama constitutes an appropriate model to investigate functional morphology, especially under ongoing climate warming.

In this study, the morphological characteristics and locomotion (running and burrowing) performance in different soil matrices of *P. przewalskii* from the Gegentala and Shierliancheng populations were compared. The objective was to determine the correlation between morphology and function in locomotor performance. As longer limbs could increase lizard sprint speed in open habitats and longer claws may enhance burrowing capacity in burrow‐digging lizards, it was hypothesized that on more solid and open soil substrates, lizards would possess longer limbs and claws (or digits) to promote mobility and burrowing ability.

## 2 Materials and Methods

### 2.1 Ethics Approval and Consent to Participate

Ethics approval for the animal collection, handling, and husbandry was given by Animal Ethics Committees at Wenzhou University (WZU‐038).

### 2.2 Study System and Lizard Collection

The toad‐headed agama lizards exhibit a preferred temperature range of 33.9°C–39.2°C (mean 36.6°C) (Li, Wang, et al. 2017). They excavate their own burrows as predator refugia and thermal retreats from extremely high temperatures (Zeng et al. 2016). Lizards were collected from two populations, Gegentala (41°47′ N, 111°48′ E; 1390 m) and Shierliancheng (40°12′ N, 111°07′ E; 1036 m), in Inner Mongolia in July 2017 after the breeding season. All lizards were individually marked with a temporary nontoxic marker pen and released at their original capture location after the experiment. Gegentala is located north of the Yin Mountains (Figure 1a) and is a typical agricultural and pastoral area (average annual temperature: 3.4°C and average annual precipitation: 200–320 mm). The soil matrix mainly consists of chestnut soil and sandy loam (Figure 1b). The dominant plant in Gegentala is the herbaceous flowering plant of the genus *Chenopodium*, which is primarily influenced by rainfall. In contrast, Shierliancheng is located south of the Yin Mountains (Figure 1a) and is a typical desert steppe undergoing serious soil desertification (average annual temperature: 7.8°C and average annual precipitation: 200–380 mm). The soil matrix comprises eolian sandy soil (Figure 1c), and the dominant plant is *Artemisia ordosica*. Under climate warming, the precipitation level in Gegentala is predicted to decrease (current: 325 mm, 2050: 248 mm, 2070: 250 mm under RCP 4.5), whereas that in Shierliancheng is predicted to increase (current: 431 mm, 2050: 466 mm, 2070: 461 mm under RCP 4.5) (https://www.worldclim.org).

### 2.3 Microhabitat Characteristics

Five transects were set up in both Gegentala and Shierliancheng in July 2017 to determine microhabitat characteristics. The transects were 250 m long, with an interval of 500 m between two adjacent transects. A 1 × 1 m^2^ plot was placed at 50 m intervals along the transect to sample vegetation characteristics. Totally, 25 sample plots for each population were set to record vegetation cover, height (±0.1 cm), and concealment. To measure vegetation concealment, a lizard model was placed at the center of the plot, and the invisible proportion of the model was estimated from each of the four corners at a distance of 5 m from the center point. The concealment for each plot is the average of these four values. The characteristics of the soil matrix were then determined at each sample. Soil from each sample plot (at a depth of 5–15 cm) was collected, dried, and filtered through a sieve (1 × 1 mm). The percentage of sand particles was calculated using Equation (1):

(1) $$\begin{array}{c} \text{Sand particle rate}\;\left(\%\right)=\\ \left(\mathrm{dry}\;\text{mass of soil}–\mathrm{dry}\;\text{mass of coarse particles}\right)/\mathrm{dry}\;\text{mass of soil} \end{array}$$

Seventy‐five burrows from each population were randomly searched for the soil hardness test on sunny days. The soil hardness of lizard burrows at four depths (i.e., 1, 5, and 15 cm deep from the surface and at the bottom of the burrow) was measured with a soil hardness tester (0.25 kg/cm^2^, SL‐TYD, Shandong, China) in Gegentala and Shierliancheng.

### 2.4 Morphological Measurement

**FIGURE 2:**
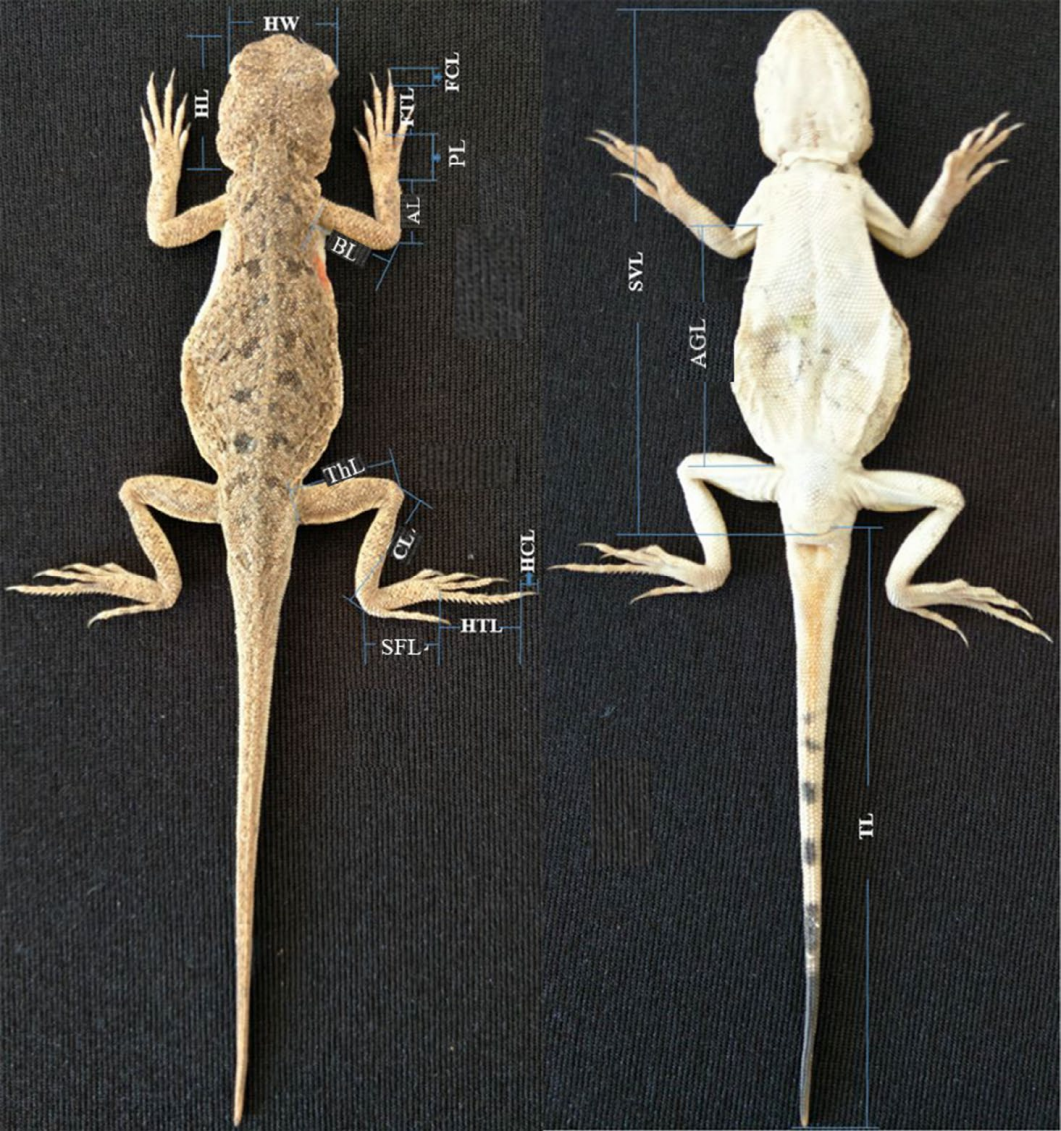
Morphological indices of *Phrynocephalus przewalskii* . AGL, axillar–groin length; AL, antebrachium length; BL, brachium length; CL, crus length; FCL, front claw length; FTL, front toe length; HCL, hind claw length; HL, head length; HTL, hind toe length; HW, head width; PL, palm length; SFL, sole foot length; SVL, snout–vent length; ThL, thigh length; TL, tail length.

A total of 124 (67 females, 57 males) and 92 (51 females, 41 males) adult toad‐headed agamas ( *P. przewalskii* ) were collected from the Gegentala and Shierliancheng populations, respectively. The morphometric measurements for each population were recorded using digital Vernier calipers (±0.01 mm, Mitutoyo 500, Japan). All measurements were taken from the right side. The indices of morphology included snout–vent length (SVL), axillar–groin length (AGL), tail length (TL), head length (HL), head height (HH), head width (HW), brachium length (BL), antebrachium length (AL), palm length (PL, measured from the wrist to the base of the longest toe), front toe length (FTL, length of the longest toe of the forelimb not including the claw), front claw length (FCL, length of the claw of the front toe), thigh length (ThL), crus length (CL), sole foot length (SFL, measured from the ankle to the base of the longest toe), hind toe length (HTL, length of the longest toe of the hindlimb not including the claw), and hind claw length (HCL, length of the claw of the hind toe) following Wu et al. (2015) (Figure 2). Subsequently, the lizards were weighed (±0.001 g, Jiming, JM‐BL5003, Ningbo, China) and released to the field where they were collected.

### 2.5 Running Performance

In July 2017, 16 adult *P. przewalskii* individuals (11 females and 5 males) from each population were collected to analyze locomotion. Intact surface soil layers (~20 cm) from the lizards' habitats were excavated using flat shovels. After collection, the lizards and the soil samples were transferred to the field station, where the lizards were kept under common conditions as follows: the lizards were housed in terraria (500 × 400 × 350 mm, length × width × height) in a temperature‐controlled room at 20°C. A 40‐W heating lamp was set above one end of each terrarium to produce a thermal range of 20°C–45°C within the heat cycle from 08:00 to 18:00. Mealworms, crickets, and water were provided *ad libitum*.

After 3 days of rearing, running performance was tested at 36°C, which is the mean selected temperature of *P. przewalskii* (Li, Wang, et al. 2017). Before the test, the lizards were acclimatized to 36°C for 1 h. Running trials were conducted on a custom‐made racetrack (length × width × height, 1000 × 100 × 150 mm) horizontally on the soil matrix (5‐cm‐thick) of Gegentala and Shierliancheng. We introduced the lizard from one end of the track for each test and then stimulated the lizard dorsally with a paintbrush to run down the track. The processes were recorded using a video camera (Sony DCR‐SR220E, Japan). Each lizard was tested twice on each soil matrix in a random sequence with an interval of 1 h. The sprint speed and average speed were calculated using an AVS Video Editor software (V9.4, Online Media Technologies Ltd.). The sprint speed was calculated as the fastest speed of the lizard running over a 20 cm interval; the average speed was the average speed of the lizard running over a 1 m track (Sun et al. 2014).

### 2.6 Burrowing Performance

After sprint ability determination, the burrowing performances of the lizards were tested. The soil matrices from Gegentala and Shierliancheng were placed in plastic boxes (length × width × height, 550 × 600 × 380 mm). According to the water content of the soil in the field, we added 8 L of water to the soil and placed the boxes outdoors for 15 days. We then set antibird nets and a plastic membrane over the top of the boxes. A camera (DCR‐SR220E, Sony Corporation, Tokyo, Japan) was hung above each box to record the burrowing processes of the lizards. During the test, the boxes were exposed to a natural light cycle, with an average surface temperature of 25.5°C (11.5°C–55°C). Each lizard was introduced to the boxes to acclimate to the test surroundings overnight for 12 h, from 19:00 to 07:00. The following day, we started recording the burrowing behavior of the lizards for 11 h from 07:00 to 18:00. Feed and water were provided *ad libitum*. The burrowing ability tests were conducted twice in a random sequence every other day for each lizard in the boxes with Gegentala and Shierliancheng soil matrix, respectively. After the burrowing test, we measured the burrow length, width, and height following Li, Hao, et al. (2017) and recorded the duration of burrowing (burrowing time: the time interval between the initiation of substrate excavation and complete bodily submersion, excluding pause intervals) through the video. The volumes of the burrows were calculated according to the typical structure of the burrows built by *P. przewalskii* , including a long tunnel with a half ellipse cross‐section (Figure S1; Li, Hao, et al. 2017), using Equation (2):

(2) Burrow volume=1/2π×height of burrow cross−section×1/2width of burrow cross−section×length of the burrow

Burrowing speed was calculated using Equation (3):

(3) Burrowing speed=Burrow volume/Burrowing time

### 2.7 Statistical Analysis

The statistical analysis was conducted using SPSS software (version 21.0; SPSS Inc., Chicago, IL). We used a Shapiro–Wilk test for normality and Levene's test for homogeneity. Data were normalized by arcsin‐ or log transformations when necessary. An independent *t‐*test was conducted to analyze the differences in the characteristics of microhabitats between Gegentala and Shierliancheng, including vegetation traits, soil matrix, and hardness. To exclude the influence of individual size on morphological characteristics, a regression analysis of the morphological index against SVL was performed (Du et al. 2005). The residual values were then used to detect the differences between the Gegentala and Shierliancheng populations using independent *t‐*tests.

Linear mixed models were used to analyze sprint speed, average speed, burrow length, width, height, volume, burrowing time, and burrowing speed, with population, soil matrix, and their interaction as fixed factors, lizard identity as a random factor, and SVL as a covariate in the analysis of burrow width. In addition, linear regression was used to analyze the relationship between functions (i.e., sprint speed and burrowing speed tested on soil from their original region) and morphological characteristics of the lizards, in which residual values of the morphological characteristics against SVL were used. Linear regression was also used to analyze the relationships between running performance (sprint speed and average speed) and burrowing performance (burrowing time and speed). All the statistical levels of significance were set as α = 0.05.

## 3 Results

### 3.1 Microhabitat Characteristics

With the exception of vegetation coverage, all microhabitat characteristics differed significantly between Gegentala and Shierliancheng (Table 1). The vegetation height, concealment, and sand particle rate in Shierliancheng were significantly greater than those in Gegentala. The soil was harder in Gegentala than in Shierliancheng at each depth that was measured (Table 1).

**TABLE 1:** Characteristics of the microhabitats of *Phrynocephalus przewalskii* in Gegentala and Shierliancheng.

Variables	Gegentala	Shierliancheng	Statistical analysis
Vegetation height (cm)^a^	12.35 ± 1.00	34.37 ± 3.93	*t* **= −6.298**, df = 48, ***p* < 0.001**
Vegetation coverage (%)	64.52 ± 5.04	53.68 ± 4.94	*t* = 1.535, df = 48, *p* = 0.131
Vegetation concealment (%)^b^	53.25 ± 6.08	89.79 ± 1.50	**t = −5.974**, df = 48, ***p* < 0.001**
Sand particles rate (%)^b^	79.12 ± 0.46	93.26 ± 0.30	**t = −25.825**, df = 48, ***p* < 0.001**
Soil hardness (kg/cm^3^)			
1‐cm depth^a^	11.43 ± 0.90	0.84 ± 0.04	**t = 46.023**, df = 148, ***p* < 0.001**
5‐cm depth^a^	15.05 ± 0.40	2.56 ± 0.12	**t = 36.291**, df = 148, ***p* < 0.001**
15‐cm depth^a^	10.86 ± 0.36	4.35 ± 0.19	**t = 17.926**, df = 148, ***p* < 0.001**
Bottom^a^	9.66 ± 0.35	4.57 ± 0.16	**t = 14.233**, df = 148, ***p* < 0.001**

*Note:* Data are shown as mean ± standard error. The significance level is set at α = 0.05 and is shown in bold font.

^a^ Log‐transformed data, and hashes.

^b^ Arc‐transformed data.

### 3.2 Morphological Characteristics

The axillar–groin length, head height, brachium length, antebrachium length, palm length, front toe length, front claw length, and hind claw length of lizards from Gegentala were significantly higher than those of lizards from Shierliancheng (Table 2). The tail length of the lizards from Gegentala was significantly shorter than that of lizards from Shierliancheng (Table 2). Other characteristics, including body mass, head length, head width, thigh length, crus length, sole foot length, and hind toe length, did not significantly differ between the two populations (Table 2).

**TABLE 2:** Morphological characteristics of *Phrynocephalus przewalskii* in Gegentala and Shierliancheng.

Morphology	Northern population	Southern population	Statistical analysis
	*n* = 124	*n* = 92	
Body mass (g)	4.47 ± 0.07	4.25 ± 0.09	*t* = 1.848, df = 214, *p* = 0.066
Snout–vent length (mm)	49.75 ± 0.24	49.24 ± 0.34	*t* = 1.245, df = 214, *p* = 0.214
Axillar–groin length (mm)	24.99 ± 0.22	23.98 ± 0.26	***t* = 2.713, df = 214, *p* = 0.007**
Tail length (mm)	56.17 ± 0.44	62.35 ± 0.48	*t* = −10.692, df = 214, *p* **< 0.001**
Head length (mm)	12.15 ± 0.09	12.21 ± 0.08	*t* = −1.016, df = 214, *p* = 0.311
Head height (mm)	6.99 ± 0.04	6.79 ± 0.05	***t* = 2.644, df = 214, *p* = 0.009**
Head width (mm)	10.66 ± 0.07	10.59 ± 0.08	*t* = −0.128, df = 214, *p* = 0.898
Brachium length (mm)	9.83 ± 0.10	8.77 ± 0.10	***t* = 7.260, df = 214, *p* < 0.001**
Antebrachium length (mm)	7.52 ± 0.06	7.11 ± 0.07	***t* = 4.191, df = 214, *p* < 0.001**
Palm length (mm)	3.83 ± 0.04	3.38 ± 0.05	***t* = 7.199, df = 214, *p* < 0.001**
Front toe length (mm)	6.17 ± 0.10	5.72 ± 0.08	***t* = 3.219, df = 214, *p* = 0.001**
Front claw length (mm)	2.68 ± 0.03	2.38 ± 0.03	***t* = 5.930, df = 214, *p* < 0.001**
Thigh length (mm)	11.00 ± 0.08	10.91 ± 0.08	*t* = 0.414, df = 214, *p* = 0.679
Crus length (mm)	13.05 ± 0.08	12.93 ± 0.10	*t* = 0.462, df = 214, *p* = 0.645
Sole foot length (mm)	5.90 ± 0.07	6.02 ± 0.07	*t* = −1.126, df = 214, *p* = 0.261
Hind toe length (mm)	8.79 ± 0.10	8.99 ± 0.11	*t* = −1.555, df = 214, *p* = 0.121
Hind claw length (mm)	2.68 ± 0.04	2.53 ± 0.03	***t* = 2.865, df = 214, *p* = 0.005**

*Note:* Data are shown as mean ± standard error. The significance level is set at *α* = 0.05 and is shown in bold font.

### 3.3 Running Performance

**FIGURE 3:**
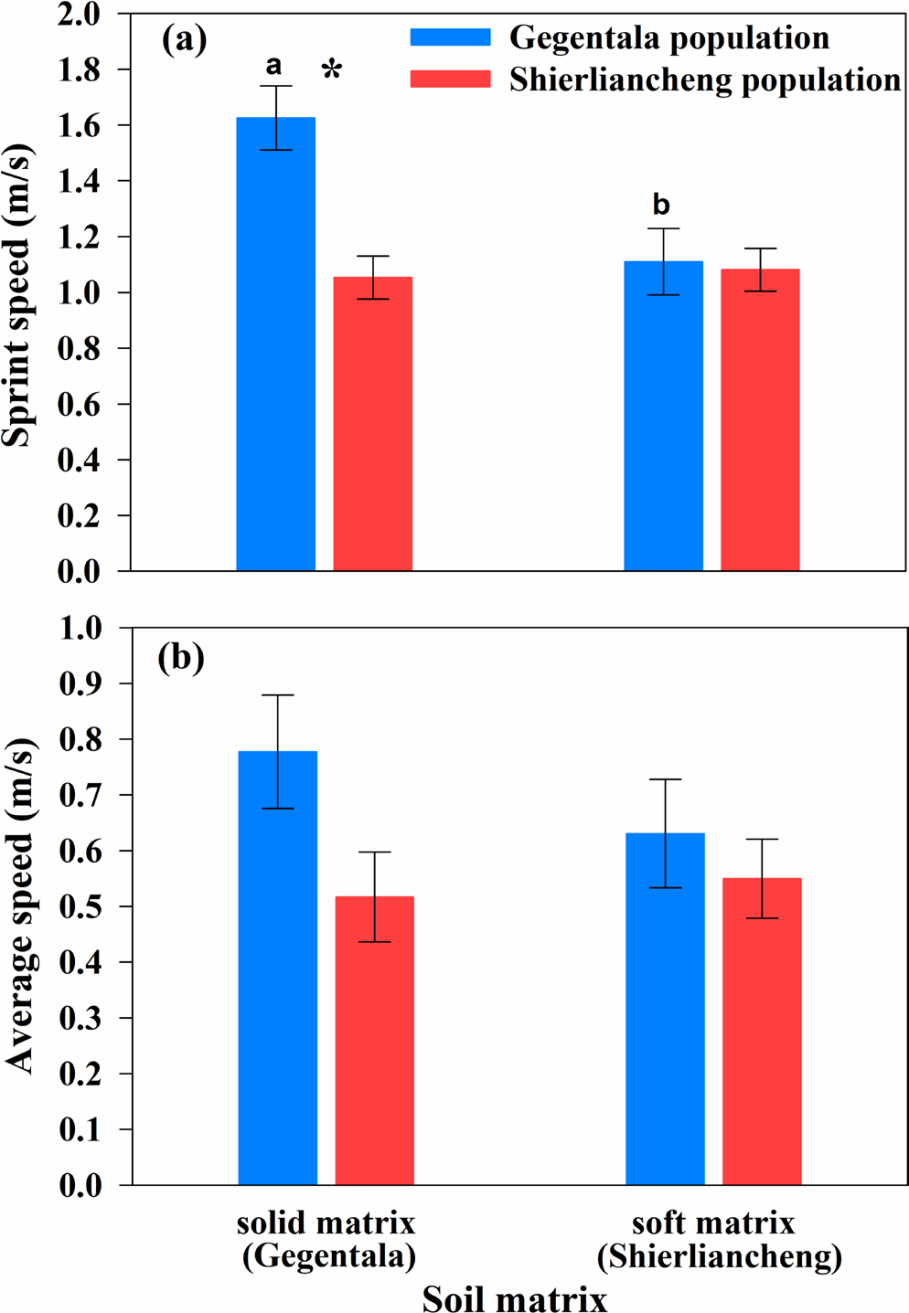
Sprint speed (a) and average speed (b) of *Phrynocephalus przewalskii* from Gegentala and Shierliancheng populations. Asterisk indicates significant differences between populations. Different lowercases indicate significant difference between soil matrices in that from Gegentala population. Data are expressed as mean ± standard error.

The sprint speed of lizards was affected by the soil matrix, the origin of the population, and the interaction between the soil matrix and the population (Table 3). The sprint speed of the lizards was higher on the solid soil matrix (i.e., soil from Gegentala) than on the soft soil matrix (i.e., soil from Shierliancheng). Lizards from Gegentala had higher sprint speeds than those from Shierliancheng. Additionally, the sprint speed of lizards from Gegentala was enhanced on the solid soil matrix compared to the sprint speed of lizards from Schierliancheng on the same soil (Table 3, Figure 3a). The average speed was not affected by the soil substrate, the origin of the population, or the interaction between the soil matrix and the population (Table 3, Figure 3b).

**TABLE 3:** Statistical analysis of locomotion and burrowing ability of *Phrynocephalus przewalskii* .

Variables	Statistical analysis		
	Soil matrix	Population	Soil matrix × population
Sprint speed (m/s)	***F***_**1,60**_ **= 5.682, *p* = 0.020**	***F***_**1,60**_ **= 8.642, *p* = 0.005**	***F***_**1,60**_ **= 7.068, *p* = 0.010**
Average speed (m/s)	*F*_1,30_ = 0.645, *p* = 0.428	*F*_**1,30**_ = 2.494, *p* = 0.125	*F*_1,30_ = 1.615, *p* = 0.214
Burrow length (cm)	***F***_**1,51**_ **= 10.148, *p* = 0.002**	***F***_**1,51**_ **= 4.731, *p* = 0.034**	*F*_1,51_ = 0.032, *p* = 0.859
Burrow width (cm)	***F***_**1,26.6**_ **= 12.913, *p* = 0.001**	*F*_1,27.2_ = 0.135, *p* = 0.853	*F*_1,26.5_ = 3.461, *p* = 0.074
Burrow height (cm)	*F*_1,51_ = 1.121, *p* = 0.295	*F*_1,51_ = 0.562, *p* = 0.457	*F*_1,51_ = 0.024, *p* = 0.878
Burrow volume (cm^3^)	*F*_1,51_ = 3.583, *p* = 0.064	***F***_**1,51**_ **= 5.545, *p* = 0.022**	*F*_1,51_ = 0.010, *p* = 0.920
Burrowing time (min)	*F*_1,26.6_ = 1.317, *p* = 0.261	*F*_1,27.2_ = 2.477, *p* = 0.127	*F*_1,26.7_ = 0.222, *p* = 0.641
Burrowing speed (cm^3^/min)	*F*_1,27.3_ = 0.001, *p* = 0.975	*F*_1,28.7_ = 0.991, *p* = 0.328	*F*_1,27.3_ = 0.108, *p* = 0.745

*Note:* The significance level is set at *α* = 0.05 and is shown in bold font.

### 3.4 Burrowing Performance

**FIGURE 4:**
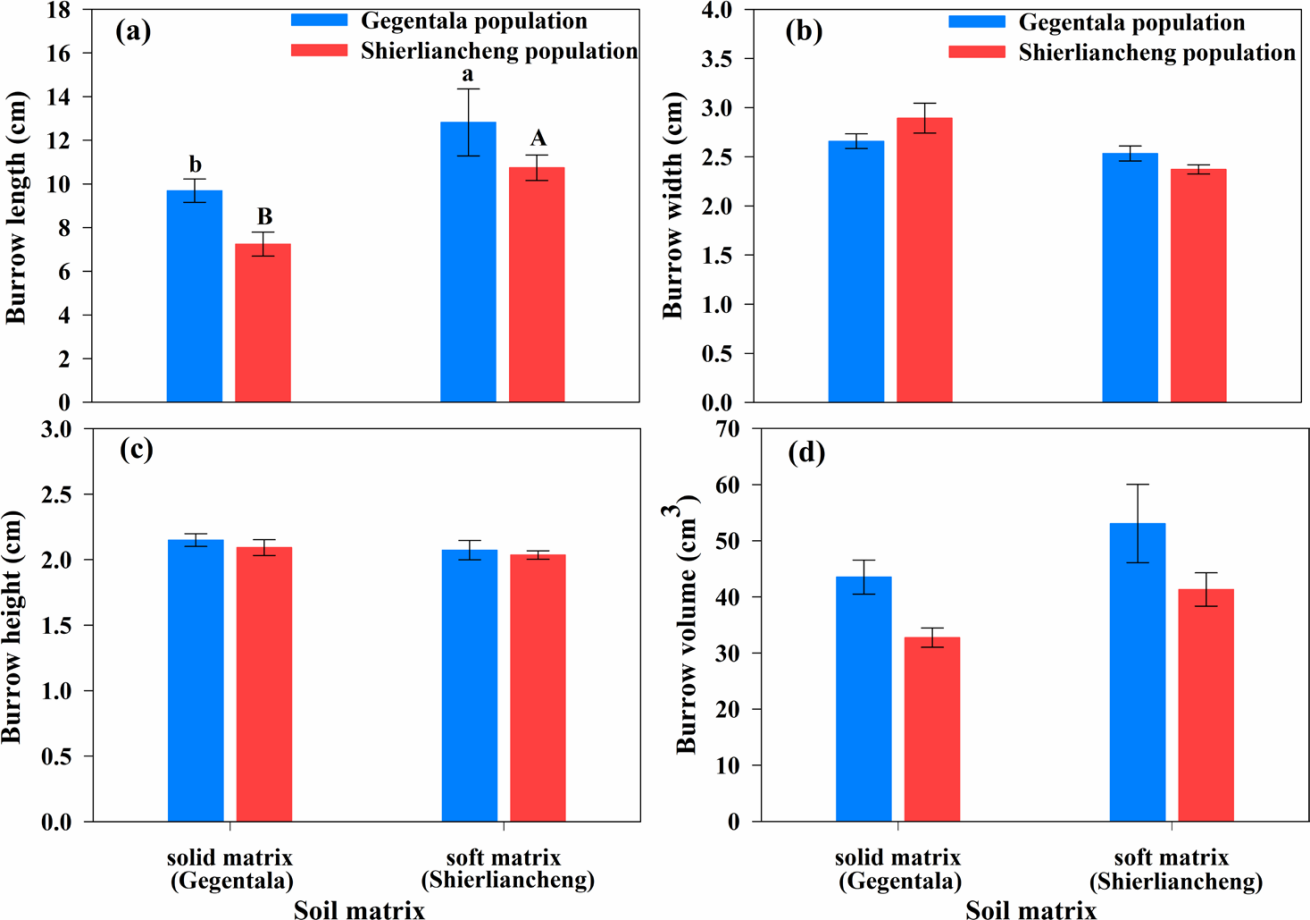
Burrowing ability of *Phrynocephalus przewalskii* from Gegentala and Shierliancheng on solid and soft soil matrices. The burrowing ability was expressed by (a) burrow length, (b) burrow width, (c) burrow height, and (d) burrow volume. Different capitals indicate significant difference between soil matrices in burrowing ability of lizards from Shierliancheng population, while different lowercases indicate significant difference between soil matrices in that from Gegentala population. Data are shown as mean ± standard error.

Twelve lizards from Gegentala and 14 from Shierliancheng burrowed successfully during the test. The length of the burrow was longer in lizards from Gegentala than in those from Shierliancheng. In addition, the burrow length was shorter in a solid soil matrix than in a soft soil matrix in both populations. However, the interaction between soil and population did not affect the burrow length (Table 3, Figure 4a). The burrow width was not affected by the origin of the population or the interaction between the soil matrix and population. However, the burrow was wider in solid than in a soft soil matrix (Table 3, Figure 4b). The burrow height was not affected by the origin of the population, the soil matrix, or the interaction between the population and soil matrix (Table 3, Figure 4c). Accordingly, the burrow volume was greater for lizards from Gegentala than for those from Shierliancheng but was not affected by the soil matrix or the interaction between the population and soil matrix (Table 3, Figure 4d). However, the burrowing time and speed were not affected by the soil matrix or the interaction between the soil matrix and the origin of the population (Table 3).

### 3.5 The Relationship Between Function and Morphological Characteristics

**FIGURE 5:**
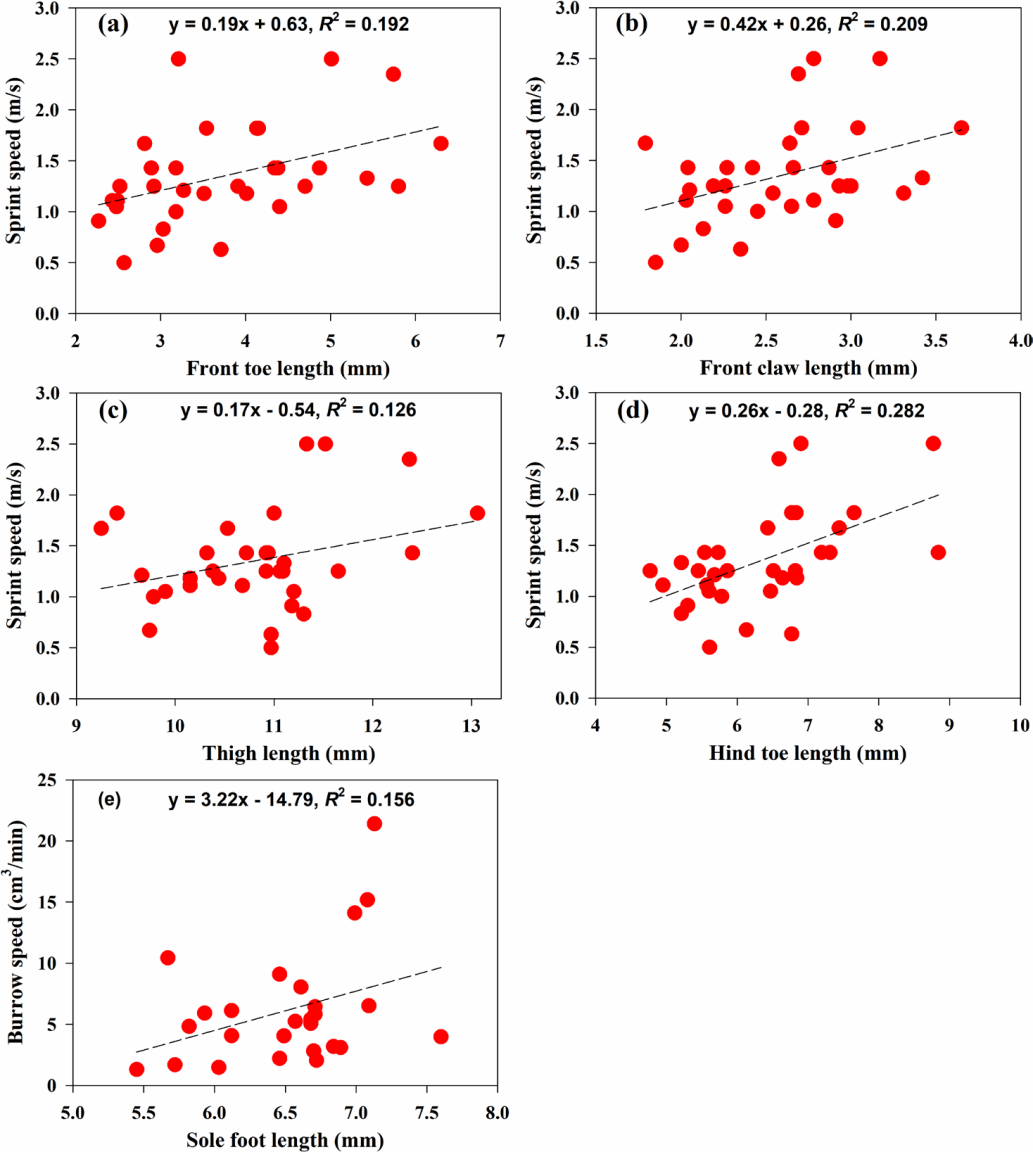
Relationship between functions (sprint speed and burrowing speed) and morphological characteristics of *Phrynocephalus przewalskii* in Gegentala and Shierliancheng, Inner Mongolia, China.

The sprint speed was positively related to front toe length, front claw length, thigh length, and hind toe length (Table 4; Figure 5a–d), while the burrowing speed was positively related to sole foot length (Table 4; Figure 5e).

**TABLE 4:** Relationship between functions (sprint speed and burrowing speed) and morphological characteristics of *Phrynocephalus przewalskii* in Gegentala and Shierliancheng, Inner Mongolia, China.

Morphology	Sprint speed	Burrowing speed
Axillar–groin length (mm)	*F*_1,30_ = 2.026, *p* = 0.165	*F*_1,24_ = 0.361, *p* = 0.553
Tail length (mm)	*F*_1,30_ = 0.413, *p* = 0.525	*F*_1,24_ = 0.006, *p* = 0.939
Head length (mm)	*F*_1,30_ = 0.165, *p* = 0.687	*F*_1,24_ = 0.018, *p* = 0.895
Head height (mm)	*F*_1,30_ = 3.600, *p* = 0.067	*F*_1,24_ = 1.377, *p* = 0.252
Head width (mm)	*F*_1,30_ = 1.994, *p* = 0.168	*F*_1,24_ = 2.039, *p* = 0.166
Brachium length (mm)	*F*_1,30_ = 4.043, *p* = 0.053	*F*_1,24_ = 0.535, *p* = 0.472
Antebrachium length (mm)	*F*_1,30_ = 1.348, *p* = 0.255	*F*_1,24_ = 0.073, *p* = 0.789
Palm length (mm)	*F*_1,30_ = 1.175, *p* = 0.287	*F*_1,24_ = 2.108, *p* = 0.159
Front toe length (mm)	***F***_**1,30**_ **= 7.109, *p* = 0.012**	*F*_1,24_ = 1.089, *p* = 0.307
Front claw length (mm)	***F***_**1,30**_ **= 7.932, *p* = 0.009**	*F*_1,24_ = 0.039, *p* = 0.884
Thigh length (mm)	***F***_**1,30**_ **= 4.316, *p* = 0.046**	*F*_1,24_ = 0.823, *p* = 0.373
Crus length (mm)	*F*_1,30_ = 2.804, *p* = 0.104	*F*_1,24_ = 0.047, *p* = 0.830
Sole foot length (mm)	*F*_1,30_ = 1.552, *p* = 0.222	***F***_**1,24**_ **= 4.433, *p* = 0.046**
Hind toe length (mm)	***F***_**1,30**_ **= 11.808, *p* = 0.002**	*F*_1,24_ = 1.107, *p* = 0.303
Hind claw length (mm)	*F*_1,30_ = 2.153, *p* = 0.153	*F*_1,24_ = 0.081, *p* = 0.778

*Note:* The significance level is set at *α* = 0.05 and is shown in bold font.

### 3.6 The Relationship Between Running and Burrowing Performances

No significant relationship was found between running and burrowing performances (All *p* > 0.272; Table S1).

## 4 Discussion

In this study, differences in the soil matrix, characteristics of vegetation, and the critical functions of locomotion and burrowing ability in two conspecific populations of toad‐headed agama from two regions in Inner Mongolia (Gegentala and Shierliancheng) were analyzed. The microhabitat of Gegentala was more open, with a more solid soil matrix than that of Shierliancheng, which has a soft soil matrix (Figure 1; Table 1). Correspondingly, lizards from Gegentala had longer limbs (Table 2), with a higher sprint speed and enhanced burrowing abilities compared with lizards from Shierliancheng (Figures 3 and 4). The positive correlation between function and morphological characteristics implies that morphology might determine the locomotion and burrowing ability. This correlation enhances our understanding of morphological strategies in response to microhabitat variations in lizards, which suggests that the morphological characteristics of the lizard are functionally compatible with the specific habitat environment (Da Silva et al. 2014).

### 4.1 Morphological Differences Between Populations

Morphological specializations, such as curly tails and stretchable appendages (fingers, feet, and claws), are adaptive to environmental variations (Sustaita et al. 2013). In the current study, the lizards from Gegentala, where the soil matrix is solid, showed longer SVL, axillar–groin length, and appendage length but shorter tail length than those from Shierliancheng (Table 2). Positive correlations were detected between locomotor traits (sprint speed and burrowing speed) and the lengths of limbs and appendages (Table 4). These morphological traits potentially enhance sprint speed and burrowing ability, thereby enhancing adaptation to a solid matrix microenvironment (Zani 2000). On the other hand, morphological differences between populations could also reflect plasticity to the local environments. For example, the different environments might shape lizard morphology during embryonic development (Buckley et al. 2009) or juvenile growth stages (Baxter‐Gilbert et al. 2021) in ways that affect adult morphology. Reciprocal transplant incubation experiments revealed *P. przewalskii* hatchlings from Gegentala were smaller than those from Shierliancheng incubated under both sites, which contrasted with the pattern observed in adults (Table 1), indicating the morphology variation between populations arising during growth stages (Hao et al. 2021).

### 4.2 Locomotor Performances

In the current study, the sprint speed of lizards from Gegentala was further enhanced on a solid soil matrix, which is akin to what is normally found in their microhabitats (Figure 3a), with a significant correlation with greater front toe length, front claw length, thigh length, and hind toe length (Tables 3 and 4), implying suitability between the soil matrix and morphology in determining the sprint speed (Kawecki and Ebert 2004). Longer limbs in the lizard increase the possibility of avoiding predators in open habitats by enhancing the sprint speed (Garland Jr. and Losos 1994; Sparkman et al. 2018). The current study also showed that the softer the soil matrix, the easier it was for lizards to burrow (Figure 4). Similarly, a previous study found that the hardness of the soil matrix significantly affected burrowing ability and burrowing time in worm lizards (Kirchner et al. 2025) and mammals (Wang et al. 2000).

In contrast to the well‐documented sprint–stamina tradeoffs in lacertid and agamid lizards (Tan et al. 2020; Vanhooydonck et al. 2014), our study found no evidence of a tradeoff between running and burrowing performance in *P. przewalskii* . This could be explained by integrative morphological mechanisms underlying both performances. For instance, stronger muscles can provide greater energy output, thereby improving running speed and burrowing ability simultaneously (Irschick and Jayne 1999; Lowie et al. 2018; Turnbull et al. 2023; Vassallo et al. 2021). Furthermore, because of its positive correlation with the maximum adhesion force (mechanical traction), the morphology of the claw is considered one of the most important factors in determining both running and burrowing ability of lizards (D'Amore et al. 2018; Tulli et al. 2011; Turnbull et al. 2023). Long claws can provide functional advantages for both burrowing (Vassallo et al. 2021; Warner et al. 2006) and sprinting (D'Amore et al. 2018; Teixeira‐Filho et al. 2001). For example, claws can cling to rough substrates and increase attachment and grasping capabilities during movement (Foster and Higham 2012; Turnbull et al. 2023; Zani 2000). In addition, locomotion is affected by the characteristics of the forelimbs (brachium and antebrachium) and hindlimbs (thigh and crus). The forelimbs and hindlimbs are mainly involved in the movement, and the stride range of the brachium may be greater than that of the thigh during running (Foster and Higham 2012; Irschick and Jayne 1999). For example, a study on *Anolis carolinensis* showed that the increase in limb flexion, stride frequency, and duty factor may be caused by an increase in the frequency of steps, which may lead to differences in the locomotor performance among different geographic populations (Foster and Higham 2012). Furthermore, this study reveals a positive correlation between hindlimb (foot) length and burrowing speed, potentially mediated through enhanced thrust generation or increased pedal surface area improving excavation efficiency. These findings align with established biomechanical hypotheses (Teixeira‐Filho et al. 2001; Ribas et al. 2004) and demonstrate evolutionary convergence with burrowing adaptations documented in mammals (Vassallo et al. 2021). Conversely, interspecific comparisons among *Ctenophorus* dragon lizards revealed that burrower species exhibit reduced limb proportions, which indicated shortened limb levers enhance force generation during substrate displacement (Thompson and Withers 2005). The limited empirical evidence regarding how morphological variation mediates burrowing performance in reptiles underscores a critical knowledge gap. Focused investigations into the biomechanical determinants of fossorial performance are essential to advance ecomorphological theory.

## 5 Conclusions

In this study, we found the *Phrynocephalus przewalskii* inhabiting open microhabitats with solid substrates exhibit longer forelimbs (brachium and antebrachium) and appendages (metacarpus, phalanges, front claw, and hind claw). They concurrently presented higher sprint speeds and enhanced burrowing capabilities, particularly on a hard soil matrix. Therefore, the differences in morphology and function between the populations support the hypothesis that morphological characteristics are compatible with locomotor performance.

## Author Contributions

**Shuran Li:** conceptualization (lead), funding acquisition (equal), investigation (supporting), methodology (equal), validation (equal), visualization (equal), writing – original draft (equal). **Jingjing Chen:** investigation (equal), methodology (equal), visualization (equal), writing – original draft (equal). **Xixi Liang:** investigation (equal), methodology (equal), visualization (equal). **Xin Hao:** investigation (equal), methodology (equal). **Chenxu Wang:** investigation (equal), methodology (equal), visualization (equal). **Baojun Sun:** investigation (supporting), resources (equal), writing – review and editing (equal). **Yongpu Zhang:** funding acquisition (lead), investigation (supporting), project administration (lead), resources (equal), supervision (equal), writing – review and editing (equal).

## Conflicts of Interest

The authors declare no conflicts of interest.

## Supporting information


**Figure S1:** Diagram of the structure of one typical burrow of *Phrynocephalus przewalskii* .
**Table S1:** The relationship between running performance (sprint speed and average speed) and burrowing performance (burrowing time and speed) of *Phrynocephalus przewalskii* in Gegentala and Shierliancheng, Inner Mongolia, China.

## Data Availability

The data that support the findings of this study are available in “dryad” at https://doi.org/10.5061/dryad.w9ghx3g1z.
