# Supplementary material for: Morphological Variations in Toad‐Headed Agama: Potential Responses to Diverse Microhabitats
Source: Ecol Evol. 2025 Sep 24;15(9):e72188. doi: 10.1002/ece3.72188 (PMC12457803; doi:10.1002/ece3.72188)
Supplement: Supplementary file 1 — Figure S1: Diagram of the structure of one typical burrow of Phrynocephalus przewalskii . Table S1: The relationship between running performance (sprint speed and average speed) and burrowing performance (burrowing time and speed) of Phrynocephalus przewalskii in Gegentala and Shierliancheng, Inner Mongolia, China. [file ECE3-15-e72188-s001.docx]

**Supporting information**

Figure S1. A diagram of the structure of one typical burrow of *Phrynocephalus przewalskii*.


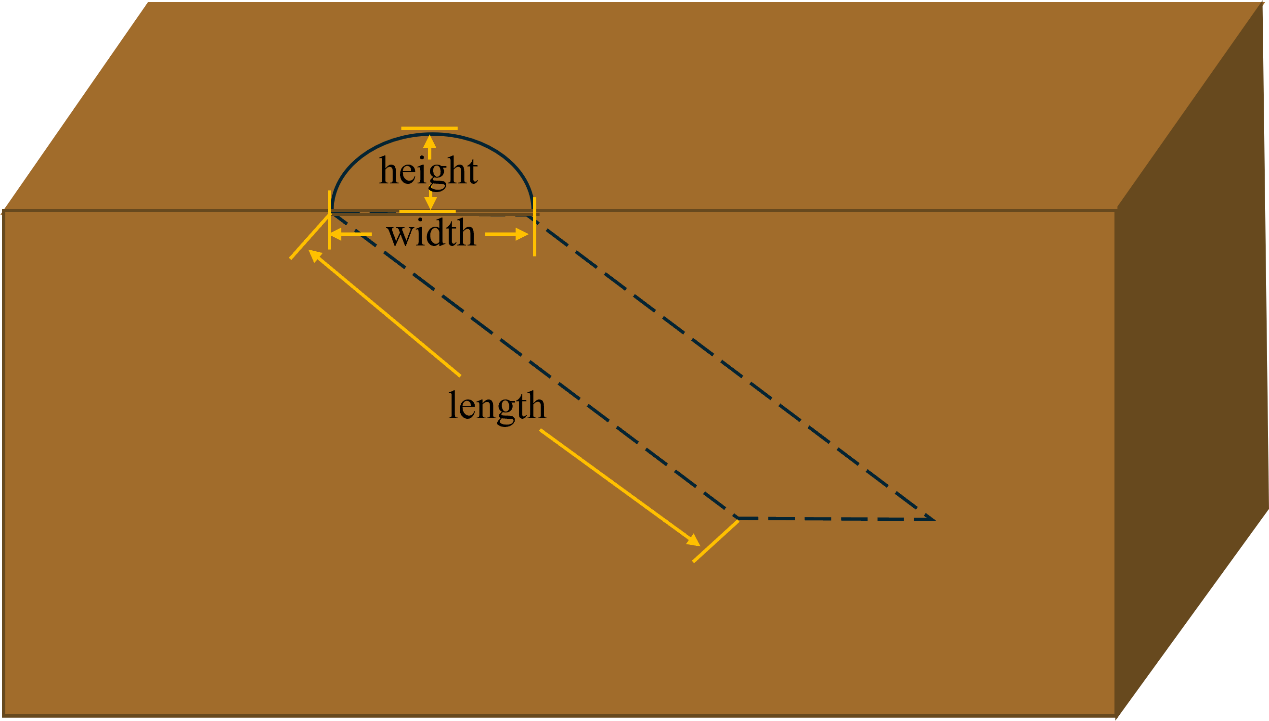


**Table S1.** The relationship between running performance (sprint speed and average speed) and burrowing performance (burrowing time and speed) of *Phrynocephalus przewalskii* in Gegentala and Shierliancheng, Inner Mongolia, China.

|  |  | Burrowing performance | |
| --- | --- | --- | --- |
|  |  | Burrowing time | Burrowing speed |
| Running performance | Sprint speed | *F*_1,24_ = 0.001, *P* = 0.977 | *F*_1,24_ = 0.024, *P* = 0.879 |
|  | Average speed | *F*_1,24_ = 1.267, *P* = 0.272 | *F*_1,24_ = 0.949, *P* = 0.340 |

The significance level is set at *α* = 0.05.
